# Identification of Circulating miRNAs Differentially Regulated by Opioid Treatment

**DOI:** 10.3390/ijms18091991

**Published:** 2017-09-16

**Authors:** Kaoru Toyama, Naoki Kiyosawa, Kenji Watanabe, Hitoshi Ishizuka

**Affiliations:** 1 Clinical Pharmacology Department, Daiichi Sankyo Co., Ltd., 1-2-58, Hiromachi, Shinagawa, Tokyo 140-8710, Japan; toyama.kaoru.fb@daiichisankyo.co.jp (K.T.); ishizuka.hitoshi.h8@daiichisankyo.co.jp (H.I.); 2 Biomarker Research Department, Daiichi Sankyo Co., Ltd., 1-2-58, Hiromachi, Shinagawa, Tokyo 140-8710, Japan; watanabe.kenji.rv@daiichisankyo.co.jp

**Keywords:** hydromorphone, oxycodone, μ-opioid receptor, microRNA, biomarker, qRT-PCR

## Abstract

Emerging evidence demonstrates functional contributions of microRNAs (miRNAs) to μ-opioid receptor (MOR) signaling, but the information so far has been mostly limited to their intracellular regulatory mechanisms. The present study aimed to investigate changes in plasma miRNA profiles elicited by opioid treatment in blood samples collected from clinical studies. Healthy male subjects were orally administered with hydromorphone or oxycodone and blood samples were collected at a specified time after the drug treatment. A total of 179 plasma miRNAs were measured using multiplex qRT-PCR. Nine and seventeen miRNAs were commonly upregulated (let-7a-5p, miR-423-3p, miR-199a-3p, miR-146a-5p, miR-23b-3p, miR-24-3p, miR-221-3p, miR-223-3p, and miR-146b-5p) and downregulated (miR-144-3p, miR-215, miR-363-3p, etc.), respectively, following opioid treatment. The MOR signaling-associated miRNAs, namely let-7 family miRNAs (i.e., let-7d-5p, let-7f-5p, let-7c, let-7e-5p), miR-103a-3p, miR-339-3p, miR-146a-5p, miR-23b-3p, miR-23a-3p, and miR-181a-5p, were differentially expressed following drug treatment. These differentially expressed miRNAs are circulating biomarker candidates that can be used to evaluate MOR stimulation and serve as novel clinical diagnostic tools for improving clinical outcomes.

## 1. Introduction

Opioids are the most widely used analgesics for the treatment of pain and related disorders. Four distinct opioid receptors have been reported, namely, μ, κ and δ receptors and opioid receptor-like-1 receptor, all of which are classified as G-protein coupled proteins [1]. μ-opioid receptor (MOR) is primarily responsible for opioid analgesia and is encoded by the OPRM1 gene, which can produce various alternatively spliced variants in humans [2]. In the past decade, opioids have been increasingly used for pain management; however, their widespread use has been accompanied by increased drug abuse, overdose, and even death [3]. Although opioids are generally effective analgesics for chronic pain, individual variability exists among patients in terms of efficacy and side effects, thereby increasing the demand for personalized medication strategies to maximize patient benefit and cost-effectiveness [3]. However, in the clinical setting, very limited options are currently available for effective patient stratification to identify individuals who are expected to optimally benefit from opioid treatment. Imaging technology represents one of the most promising tools that can be applied to investigate the functional dynamics in the central nervous system (CNS) in response to opioid treatment in males [4,5] and is particularly expected to be useful for analgesic drug development. However, measurable biomarkers using easily accessible biological specimens, such as blood, are desired for practical medical use.

MicroRNAs (miRNAs) represent a class of non-coding small RNAs with average lengths of 22-nucleotide and are produced by the RNase III proteins Drosha and Dicer [6]. Mature single-strand miRNA is incorporated into RNA-induced silencing complex, which guides the complex into complementary 3′- or 5′-untranslated regions (UTRs), open reading frames, or promoter regions of target genes [7]. miRNAs generally act as negative regulators of target gene products via mRNA cleavage or translational repression, but in some cases, miRNAs can also upregulate target genes [8]. It is now widely recognized that miRNAs play crucial roles in almost all aspects of developmental and pathological processes, and disruptions in miRNA regulation are associated with certain types of cancers, liver and cardiovascular diseases, and CNS impairments, including schizophrenia and drug addiction [9,10].

Besides its functional importance, miRNAs also exhibit advantageous characteristics as biomarkers for clinical use [11]. In particular, miRNAs are remarkably stable in body fluids, such as blood, and even under varying pH conditions, boiling, multiple freeze-thaw cycles, and extended storage [12]. The high stability of miRNAs in the blood is attributed to binding to Argonaute 2 protein or incorporation to microvesicles, such as exosomes, which protect miRNAs from degradation under RNase-rich circulation conditions [13]. Exosomes are small vesicles with diameters ranging from 30–120 nm that are present in most biological fluids. Exosomes act as carriers of various molecules, such as proteins, lipids, mRNAs, and miRNAs [14] and can cross the blood-brain barrier [15]. A number of reports have revealed that certain miRNAs can be secreted by tumor cells [7]. Studies have also demonstrated that corresponding exosome miRNA profiles of tumor cells can reflect the intracellular miRNA content, although whether the selectivity of miRNAs embedded into exosomes is strictly regulated remains to be clarified [16].

Previous studies have demonstrated that various miRNAs, such as let-7 family of miRNAs and miR-23b, are functionally associated with the endogenous MOR system and pain signaling [2]. Neurons and glial cells actively release microvesicles into the extracellular space, thereby providing evidence that exosome-mediated intracellular communication may be a common mechanism in CNS [17]. Thus, we hypothesized that the pharmacological response of CNS cells against MOR activation can alter plasma miRNA profiles, which can in turn act as potential blood biomarkers that can be used to evaluate MOR activation.

The objective of the present study was to identify plasma miRNAs that are differentially regulated upon treatment with the MOR activators hydromorphone and oxycodone [18]. Measurements were performed on blood samples previously collected from two independent clinical studies, in which extended-release (ER) tablets of hydromorphone or oxycodone were administered to healthy male volunteers [19,20].

## 2. Results

### 2.1. Overview of Differentially Regulated miRNAs

**Figure 1:**
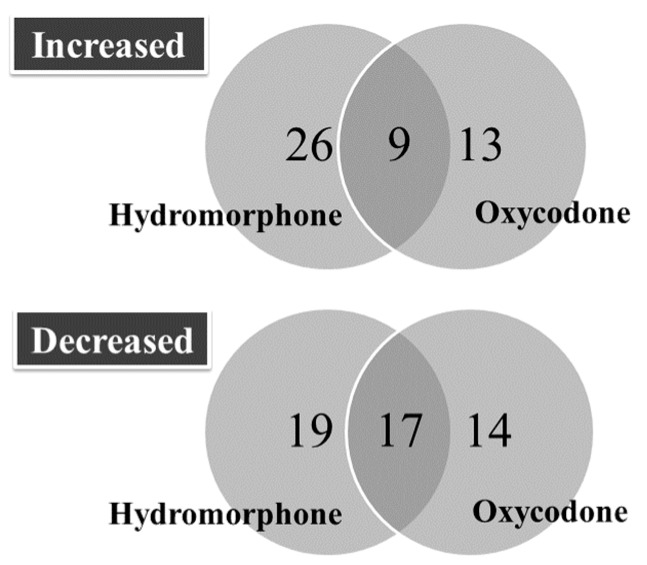
Differentially regulated miRNAs at 24 h after hydromorphone or oxycodone treatment. The number of differentially regulated miRNAs were determined for subjects treated with either hydromorphone (*N* = 6) or oxycodone (*N* = 23). All differentially regulated miRNAs are listed in Table 1 and Table 2.

The number of differentially regulated miRNAs is presented in Figure 1, and their corresponding changes in expression levels (i.e., ΔCq_pre_–ΔCq_24 h_) are presented in Table 1 and Table 2. Nine and seventeen miRNAs were found to be commonly upregulated and downregulated at 24 h following treatment with hydromorphone and oxycodone, respectively. The number of miRNAs that were commonly downregulated between hydromorphone- and oxycodone-treated subjects (47% and 55%, respectively) were higher than the number of commonly upregulated miRNAs (26% and 41% for hydromorphone and oxycodone, respectively).

### 2.2. Upregulated miRNAs upon Hydromorphone and Oxycodone Treatment

**Figure 2:**
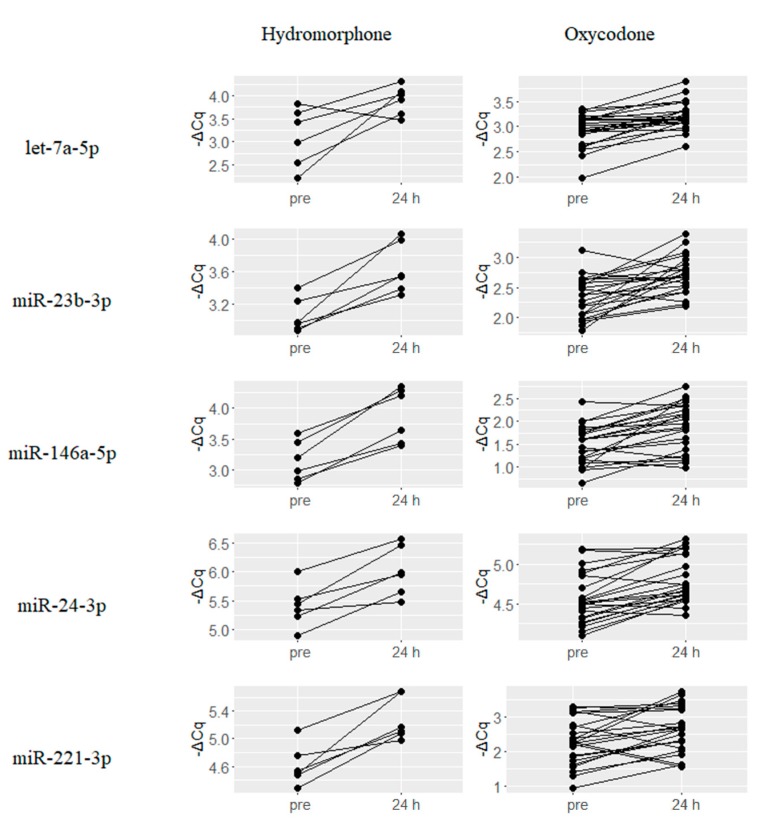
miRNAs that were commonly upregulated in response to treatment with hydromorphone and oxycodone. Nine miRNAs were commonly upregulated at 24 h after treatment with hydromorphone and oxycodone, and the five representative miRNAs are presented. ΔCq represents the normalized Cq value of target miRNAs using global mean normalization method.

Plasma levels of nine miRNAs, namely, let-7a-5p, miR-423-3p, miR-199a-3p, miR-146a-5p, miR-23b-3p, miR-24-3p, miR-221-3p, miR-223-3p and miR-146b-5p were found to be upregulated by both hydromorphone and oxycodone at 24 h after drug treatment. Changes in the expression levels of five representative miRNAs, namely, let-7a-5p, miR-23b-3p, miR-146a-5p, miR-24-3p, and miR-221-3p are presented in Figure 2. Average changes in expression levels for these miRNAs were higher in hydromorphone-treated subjects than in oxycodone-treated subjects. These nine miRNAs were found to derive from bona fide miRNA genes according to MirGeneDB search results [21].

### 2.3. Downregulated miRNAs upon Hydromorphone and Oxycodone Treatment

**Figure 3:**
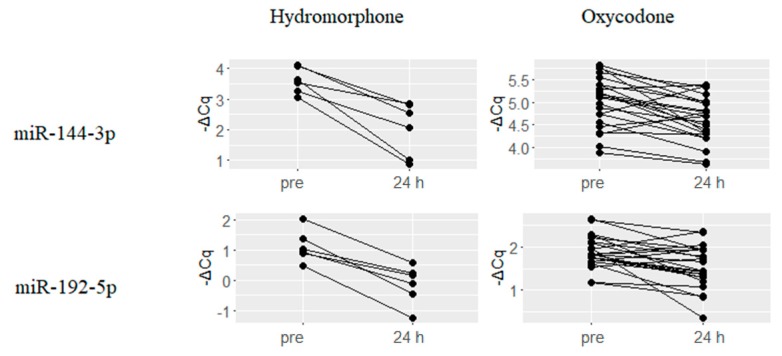

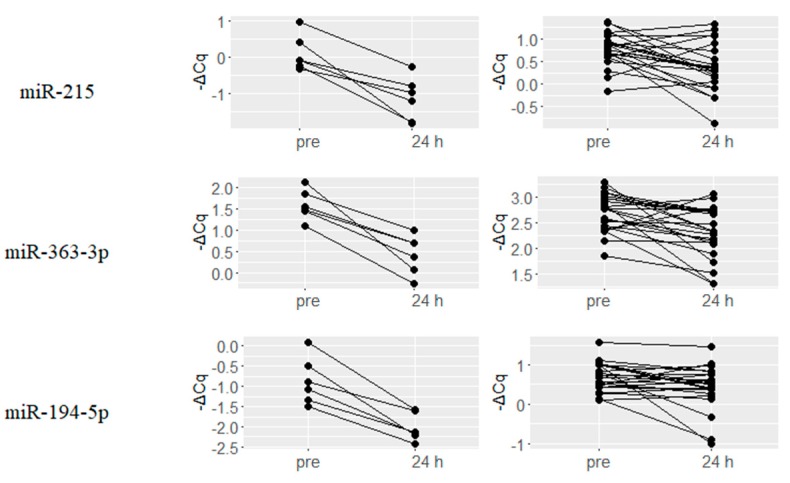
Representative miRNAs commonly downregulated upon treatment with both hydromorphone and oxycodone. Sixteen miRNAs were downregulated at 24 h after treatment with both hydromorphone and oxycodone, and the five representative miRNAs are presented. ΔCq is a normalized Cq value of target miRNAs using global mean normalization method.

A total of 17 miRNAs were downregulated upon treatment with both hydromorphone and oxycodone. Changes in the expression levels of five representative miRNAs, namely, miR-144-3p, miR-192-5p, miR-215, miR-363-3p and miR-194-5p are presented in Figure 3. Similar to the results obtained for upregulated miRNAs, hydromorphone-treated subjects showed larger average changes in expression levels than those of oxycodone-treated subjects. Among the 17 miRNAs, all the miRNAs derive from bona fide miRNA genes except for miR-451a, according to MirGeneDB search results.

### 2.4. Hydromorphone- or Oxycodone-Induced miRNA Upregulation Is Associated with μ-Opioid Receptor (MOR) Signaling

**Figure 4:**
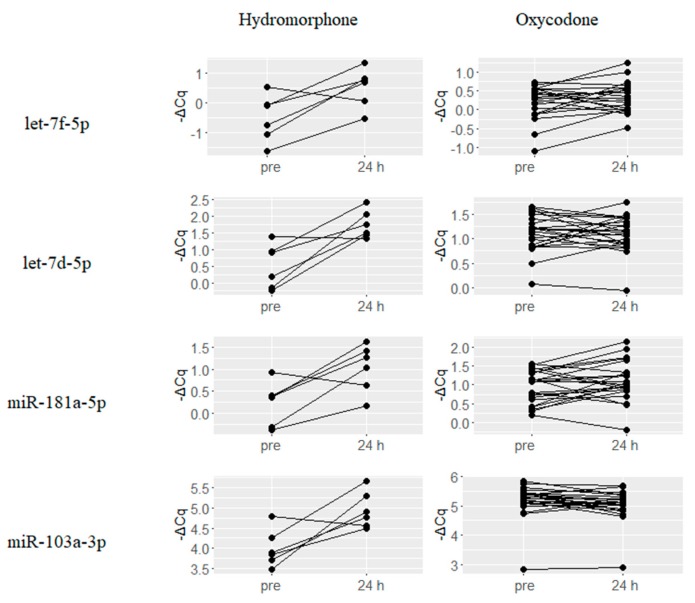
Hydromorphone-upregulated miRNAs potentially associated with μ-Opioid Receptor (MOR) signaling. Six miRNAs previously reported to be associated with MOR signaling were upregulated at 24 h after hydromorphone treatment, and representative four miRNAs are presented. ΔCq is the normalized Cq value of target miRNAs calculated using global mean normalization method.

**Figure 5:**
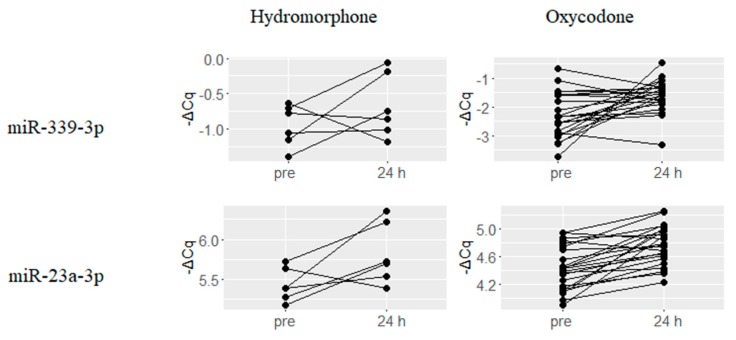
Oxycodone-upregulated miRNAs potentially associated with μ-Opioid Receptor (MOR) signaling. Two miRNAs previously reported to be associated with MOR signaling showed were upregulated at 24 h after oxycodone treatment. ΔCq is a normalized Cq value of target miRNAs using global mean normalization method.

A number of miRNAs that are predicted to be involved in MOR signaling based on previous reports or in silico binding predictions, were found to be upregulated upon treatment with either hydromorphone or oxycodone. Six miRNAs let-7f-5p, let-7d-5p, let-7c, let-7e-5p, miR-181a-5p and miR-103a-3p were upregulated by hydromorphone treatment, and representative four miRNAs are presented in Figure 4. On the other hand, two miRNAs miR-339-3p and miR-23a-3p were upregulated by oxycodone treatment (Figure 5).

## 3. Discussion

Emerging evidence has revealed the functional involvement of miRNAs in MOR signaling. However, these findings are mostly limited to intracellular regulatory mechanisms. Thus, the present study aimed to identify candidate circulating biomarkers to evaluate MOR stimulation using plasma samples collected from two previously conducted independent clinical studies, in which either hydromorphone or oxycodone were administered to healthy subjects [20,21].

Nine miRNAs were commonly upregulated by both hydromorphone and oxycodone treatments (Table 1), including let 7a-5p, miR-146a-5p and miR-23b-3p. Binding of the let-7 family of miRNAs to the 3′-UTR of OPRM1 suggests their involvement in opioid tolerance [22], and therefore the observed upregulation of let-7a-5p may reflect negative feedback response against MOR stimulation. miR-146a-5p was observed to be upregulated upon morphine treatment in human monocyte-derived macrophages [23] and was reported to play a role in pain signaling [24]. A previous study demonstrated that miR-23b-3p is upregulated by morphine and is involved in the modulation of MOR signaling [25]. On the other hand, seventeen miRNAs were downregulated in both hydromorphone- and oxycodone-treated subjects (Table 2). However, their corresponding roles in MOR signaling remain to be elucidated.

**Figure 6:**
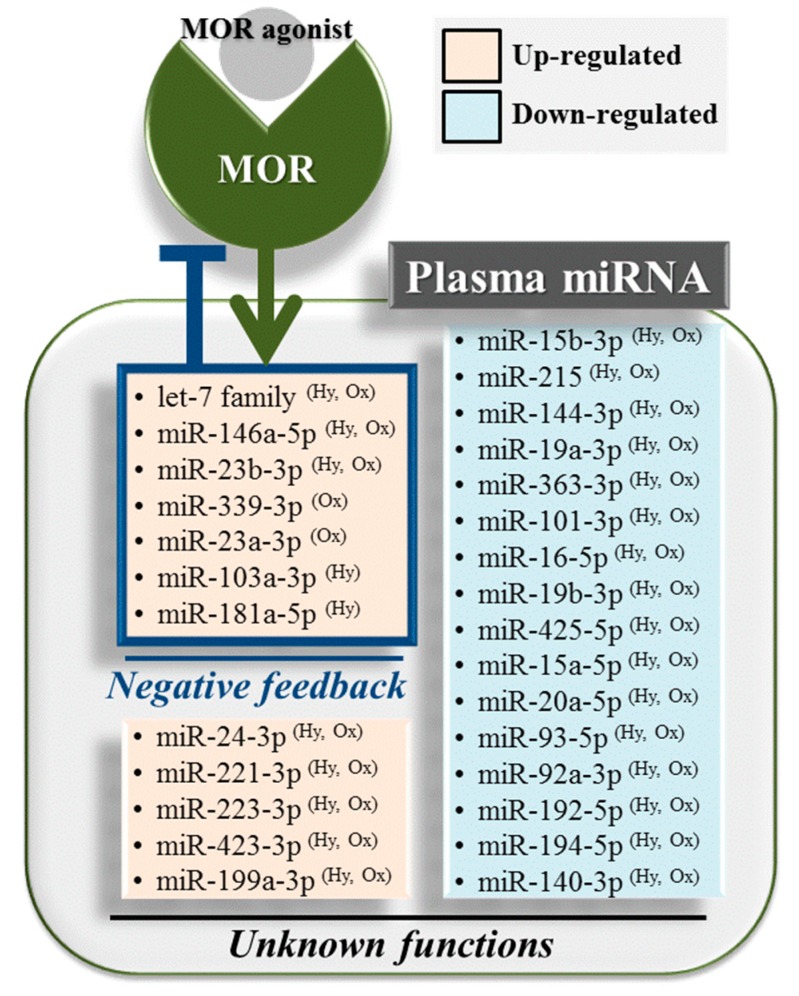
Overall miRNA expression profile following treatment with hydromorphone and/or oxycodone. miRNAs differentially regulated by MOR stimulation are summarized. miRNAs such as the let-7 family miRNAs and miR-339-3p have been reported to repress MOR function, suggesting negative feedback response against MOR stimulation. Functional involvement of the remaining differentially regulated miRNAs in MOR signaling remain to be elucidated. These miRNAs can potentially serve as candidate circulating biomarkers for evaluating MOR stimulation. miR-451a is excluded from the list of down-regulated miRNAs since it does not derive from bona fide miRNA gene. Hy and Ox represent hydromorphone and oxycodone, respectively.

In addition, several miRNAs that were reported to be functionally associated with MOR signaling were found to be differentially regulated by either hydromorphone or oxycodone. Six miRNAs, namely, let-7f-5p, let-7d-5p, let-7c, let-7e-5p, miR-181a-5p and miR-103a-3p, were upregulated upon hydromorphone treatment (Table 1). let-7 Family miRNAs (i.e., let-7f-5p, let-7d-5p, let-7c and let-7e-5p) potentially modulate MOR function as previously mentioned [22]. miR-181a-5p was found to be upregulated by morphine treatment in mouse hippocampal progenitor cell lineages, suggesting its potential role in neurogenesis [26] and drug addiction [27]. miR-103a-3p was also reported to be upregulated following chronic exposure to morphine, which is known to downregulate MOR [28]. On the other hand, oxycodone treatment resulted in the upregulation of two miRNAs, namely, miR-339-3p and miR-23a-3p (Table 1), and miR-339-3p was reported to inhibit MOR function [29]. Thus, the upregulation of these miRNAs provide evidence of negative feedback responses against MOR stimulation by hydromorphone or oxycodone. Taken together, all the aforementioned differentially regulated miRNAs are promising candidates as circulating biomarkers for evaluating MOR stimulation (Figure 6).

Our current findings provided valuable insights into MOR signaling-associated miRNA regulation. However, certain limitations need to be addressed. First, the multiplex qRT-PCR panel system used in the study does not cover a sufficient number of circulating miRNA species, considering the existence of a large number of miRNAs [30]. In future studies, miRNA-Seq analysis using next-generation sequencing technology can be used to obtain more comprehensive miRNA expression profiles; Second, additional observational time points after treatment with multiple opioids are required for a more detailed characterization of miRNA expression profiles following opioid treatment; Third, additional experiments must be conducted to validate the present findings and establish reproducibility. Although the use of miRNA-based biomarkers in diagnosis has long been expected, specificity and reproducibility in previous experiments have been generally found to be poor [10]. Indeed, the number of commonly upregulated miRNAs represented only 26% to 41% of all miRNAs upregulated in response to treatment with hydromorphone and oxycodone. One possible explanation for the low overlap is that hydromorphone and oxycodone may elicit agonist-specific pharmacological effects similar to the mechanisms by which morphine and fentanyl activate the protein kinase C and β-arrestin2 pathways following MOR stimulation, respectively [31]. Although hydromorphone acts primarily on MOR and to a lesser extent on the δ-opioid receptor, oxycodone exerts action on multiple receptors including the κ-opioid receptor [18]. Thus, although the general profiles of differentially regulated miRNAs represent the molecular dynamics associated with MOR stimulation, hydromorphone and oxycodone can also elicit drug-specific responses that can influence miRNA expression regulation. In addition, the experimental procedure and analytical methods for measurement of concentrations of circulating miRNAs have yet to be optimized in general; e.g., potential confounding factors such as variation in the results of RNA extraction and PCR and the normalization method for qRT-PCR data.

Lastly, findings from the present study may not be directly applicable to diseased subjects, since pathological conditions, such as cancer or drug addiction, are likely to considerably affect plasma miRNA profiles. For instance, let-7 family miRNAs were identified as candidate biomarkers to evaluate MOR signaling in the present study. However, let-7 miRNAs are also known to act as tumor suppressors by targeting various oncogenes [32]; let-7 miRNAs can be selectively incorporated into exosomes and secreted from tumor cells [33], resulting in elevated circulation levels. Increased circulating let-7 miRNAs can confound the interpretation of plasma let-7 expression profiles following opioid treatment. Indeed, although the present study was conducted on healthy volunteers without disease-related confounding factors, changes in the plasma let-7 level could not clearly separate subjects into the groups before and after drug treatment. These data suggest that let-7 miRNA alone will not be a practical clinical biomarker, and no other miRNAs quantified in the present study can serve as such an ideal biomarker. One potential solution is to investigate larger sets of miRNAs, such as those shown in Figure 6, instead of more specific miRNAs (e.g., let-7 family miRNAs) to capture systems-level effects (e.g., MOR stimulation). Previous studies have applied similar concepts for interpreting mRNA transcriptome data and have demonstrated that analysis using pre-specified gene sets, and not individual genes, can facilitate understanding systems-level biological impacts following drug treatment [34,35]. The accumulation of more miRNA profiling data in various diseased conditions will advance our knowledge of circulating miRNA profiles and explore their potential use in evaluating MOR signaling.

In conclusion, we identified circulating miRNAs that were differentially regulated upon hydromorphone and/or oxycodone treatment. These miRNAs serve as promising candidate biomarkers to evaluate MOR activation in the body. Further investigation will be required to better characterize these miRNAs and can facilitate the development of novel diagnostic tools to support personalized opioid medication and to improve clinical benefit and maximize cost-effectiveness of the drugs.

## 4. Materials and Methods

### 4.1. Summary of Clinical Studies

Blood samples used for miRNA profiling experiments were obtained from two previous independent clinical studies [20,21]. Both studies were conducted in accordance with the ethical principles of the Declaration of Helsinki, the guidelines on Good Clinical Practice, and locally applicable laws and regulations. The study protocol, amendments, and informed consent forms were approved by an Institutional Review Board for DS-7113 on 10 May 2013 (Showa University Karasuyama Hospital IRB, Tokyo, Japan, Clinical trial registration number: Japic-132167), and DS-5058b on 27 February 2014 (OPHAC Hospital IRB, Osaka, Japan, Japic-163475). All subjects provided written informed consent prior to study commencement.

#### 4.1.1. Hydromorphone Study (Japic-132167)

The primary objective of the study was to evaluate the safety, tolerability, and pharmacokinetics profiles following single doses of immediate-release and extended release (ER) hydromorphone tablets in healthy male Japanese subjects [19]. For miRNA measurement, blood samples were collected from a treatment group comprising six individuals administered with 6 mg of ER tablets of hydromorphone (Daiichi Sankyo Co., Ltd., Tokyo, Japan). All subjects fasted overnight (more than 10 h) and for 4 h after drug treatment. Blood samples were collected at pre-dosing and at 4, 8, 12, and 24 h after drug administration. All subjects agreed to provide blood samples for miRNA measurement. No drug-related treatment emergent adverse events (TEAEs) were identified. This study was conducted at the Clinical Research Institute for Clinical Pharmacology and Therapeutics at Showa University School of Medicine (Tokyo, Japan) in 2013.

#### 4.1.2. Oxycodone Study (Japic-163475)

The primary objective of the study was to demonstrate the bioequivalence of the newly formulated ER oxycodone tablets (Daiichi Sankyo Co., Ltd.) with a reference drug available in the market, OXYCONTIN^®^ (Shionogi Co., Ltd., Osaka, Japan) [20]. For miRNA measurement, blood samples were collected from treatment groups administered with oxycodone products containing 10 mg of oxycodone hydrochloride (*N* = 12 for each drug treatment group). All subjects fasted overnight (more than 10 h) and for 4 h after drug treatment. Blood samples were collected at pre-dosing and at 4, 9, 12, 24, and 36 h after drug treatment. One subject declined to provide blood samples for miRNA measurement, resulting in a total of 23 subjects collected for miRNA measurement. The frequencies of TEAE occurrence for subjects administered with the test and reference drugs were 12.5% and 16.7%, respectively, in which somnolence, diarrhea, nausea, proctalgia or abnormal feeling were reported. All TEAEs were relatively transient and of mild to moderate intensity and were resolved by the end of the study. This study was conducted at Osaka Pharmacology Clinical Research Hospital (Osaka, Japan) in 2014.

### 4.2. miRNA Extraction from Plasma and qRT-PCR Analysis

Experiments were conducted at Takara Bio Inc. (Shiga, Japan). miRNA extraction was conducted using the plasma samples collected at pre-dosing and at 24 h after treatments with both hydromorphone and oxycodone in the fasting state. Plasma samples were prepared with EDTA-2K, and small RNAs were extracted from 200 μL of plasma using the miRNeasy Mini Kit (Qiagen, Venlo, The Netherlands) according to the manufacturer’s instructions. Single-stranded cDNA was synthesized with Universal cDNA Synthesis Kit II, 8-64 rxns (Product No.: 203301, Exiqon, Vedbaek, Denmark). A total of 179 circulating miRNAs were measured using Serum/Plasma Focus microRNA PCR Panel (Exiqon) and LightCycler^®^ 480 Instrument II (Roche Diagnostics, Basel, Switzerland). The Cq value, which represents the PCR cycle upon reaching the designated threshold amplification level, was determined for all target miRNAs using GenEx software (Exiqon) according to the manufacturer’s instructions [36]. Target miRNAs whose amplification levels did not reach the designated threshold after the 40-cycle amplification were considered absent and were excluded from further analysis.

Cq is a logarithmic scale value, and it can be converted to linear scale according to the following formula [36]:

$$N=2^{(\Delta C}{{}^{q_{\mathrm{pre}}}}^{-}{{}^{\Delta C}}^{q_{24\;h}}{}^{)}$$

where ΔCq_pre_ and ΔCq_24 h_ represent ΔCq values at pre and 24 h, respectively, and *N* represents the ratio of miRNA levels between 24 h and pre.

### 4.3. Data Analysis

For normalization, Cq values for the target miRNAs were subtracted with the average Cq values across all miRNAs in the sample to obtain the corresponding ΔCq values [37]. ΔCq values for all data obtained at pretreatment and at 24 h following drug treatment were subjected to paired *t*-test analysis using Benjamini and Hochberg’s method to control the false discovery rate. *q* < 0.05 Was considered statistically significant. In the oxycodone study, given that the bioequivalence of the ER oxycodone tablet and OXYCONTIN^®^ has been demonstrated [20], qRT-PCR data for these two formulations were pooled for statistical analysis of oxycodone-regulated miRNAs.

## Figures and Tables

**Table 1:** Upregulated miRNAs at 24 h after treatment with hydromorphone or oxycodone.

Hydromorphone (*N* = 6)			Oxycodone (*N* = 23)		
miRNA	ΔCq_(24 h–Pre)_	*q*-Value	miRNA	ΔCq_(24 h–Pre)_	*q*-Value
hsa-miR-127-3p	−2.85	0.003	hsa-miR-361-3p	−1.18	0.005
hsa-miR-182-5p	−1.87	0.044	hsa-miR-208a	−0.89	0.003
hsa-miR-339-5p	−1.47	0.006	hsa-miR-339-3p	−0.69	0.027
hsa-miR-374b-5p	−1.39	0.003	hsa-miR-10b-5p	−0.62	0.002
hsa-miR-28-5p	−1.33	0.009	hsa-miR-28-3p	−0.59	0.042
hsa-miR-551b-3p	−1.33	0.008	hsa-miR-125a-5p	−0.51	0.004
hsa-let-7d-5p	−1.25	0.003	hsa-miR-342-3p	−0.49	0
hsa-miR-26a-5p	−1.15	0.009	hsa-miR-150-5p	−0.48	0.001
hsa-miR-382-5p	−1.09	0.037	**hsa-miR-146b-5p**	−0.46	0.005
hsa-let-7f-5p	−1.05	0.018	hsa-miR-197-3p	−0.40	0.01
hsa-miR-154-5p	−1.01	0.039	**hsa-miR-146a-5p**	−0.40	0.001
hsa-let-7e-5p	−1.00	0.042	**hsa-miR-23b-3p**	−0.38	0.001
hsa-miR-191-5p	−0.98	0.013	**hsa-miR-221-3p**	−0.36	0.027
hsa-miR-103a-3p	−0.95	0.004	hsa-miR-23a-3p	−0.33	0.001
**hsa-miR-423-3p**	−0.91	0.004	**hsa-miR-199a-3p**	−0.32	0.031
hsa-miR-1	−0.87	0.043	**hsa-let-7a-5p**	−0.29	0.027
hsa-miR-766-3p	−0.84	0.04	hsa-miR-29a-3p	−0.29	0.027
**hsa-let-7a-5p**	−0.80	0.009	**hsa-miR-223-3p**	−0.27	0.011
hsa-miR-181a-5p	−0.79	0.035	**hsa-miR-24-3p**	−0.25	0.001
**hsa-miR-199a-3p**	−0.77	0.003	hsa-miR-142-3p	−0.24	0.011
**hsa-miR-146a-5p**	−0.75	0.004	**hsa-miR-423-3p**	−0.23	0.038
hsa-miR-328	−0.73	0.002	hsa-miR-126-3p	−0.17	0.033
hsa-let-7c	−0.73	0.019			
**hsa-miR-221-3p**	−0.67	0.012			
hsa-miR-151a-5p	−0.67	0.009			
hsa-miR-142-5p	−0.63	0.026			
**hsa-miR-24-3p**	−0.61	0.005			
hsa-miR-324-5p	−0.60	0.035			
hsa-miR-107	−0.60	0.022			
hsa-miR-151a-3p	−0.60	0.011			
**hsa-miR-23b-3p**	−0.58	0.005			
**hsa-miR-146b-5p**	−0.56	0.035			
**hsa-miR-223-3p**	−0.52	0.02			
hsa-miR-652-3p	−0.49	0.006			
hsa-miR-140-5p	−0.41	0.043			

miRNAs upregulated upon hydromorphone and/or oxycodone treatment are presented. Underlined miRNAs are commonly upregulated between both hydromorphone and oxycodone. *q*-values are FDR adjusted *p* values of paired *t*-test between Pre and 24 h.

**Table 2:** Downregulated miRNAs at 24 h after treatment with hydromorphone or oxycodone.

Hydromorphone (*N* = 6)			Oxycodone (*N* = 23)		
miRNA	ΔCq_(24 h–Pre)_	*q*-Value	miRNA	ΔCq_(24 h–Pre)_	*q*-Value
hsa-miR-885-5p	2.47	0.011	hsa-miR-182-5p	1.34	0.004
hsa-miR-122-5p	1.71	0.001	**hsa-miR-15b-3p**	0.6	0
hsa-miR-375	1.61	0.002	**hsa-miR-451a**	0.41	0.002
**hsa-miR-144-3p**	1.58	0.003	**hsa-miR-215**	0.4	0.003
**hsa-miR-451a**	1.39	0.003	**hsa-miR-144-3p**	0.4	0.003
hsa-let-7b-3p	1.3	0.011	**hsa-miR-19a-3p**	0.39	0.001
**hsa-miR-192-5p**	1.26	0.001	**hsa-miR-363-3p**	0.39	0.003
**hsa-miR-215**	1.25	0	**hsa-miR-192-5p**	0.35	0.003
**hsa-miR-363-3p**	1.17	0.003	**hsa-miR-101-3p**	0.34	0.003
**hsa-miR-194-5p**	1.14	0.004	**hsa-miR-16-5p**	0.33	0.003
hsa-miR-660-5p	1.14	0.003	hsa-miR-106b-5p	0.33	0.003
**hsa-miR-140-3p**	1.11	0.004	**hsa-miR-19b-3p**	0.32	0.002
hsa-miR-378a-3p	1.1	0	hsa-miR-185-5p	0.32	0.005
hsa-miR-486-5p	1.08	0.006	hsa-miR-17-5p	0.29	0.038
hsa-miR-424-5p	1.02	0.003	hsa-miR-324-3p	0.29	0.006
hsa-miR-144-5p	1	0.03	**hsa-miR-425-5p**	0.29	0.004
**hsa-miR-16-5p**	1	0.003	hsa-miR-18b-5p	0.27	0.004
hsa-miR-502-3p	0.98	0.012	hsa-miR-18a-5p	0.27	0.003
hsa-miR-16-2-3p	0.97	0.011	**hsa-miR-15a-5p**	0.27	0.01
hsa-miR-99a-5p	0.95	0.004	hsa-miR-106a-5p	0.26	0.005
hsa-miR-25-3p	0.95	0.003	**hsa-miR-20a-5p**	0.26	0.01
hsa-miR-365a-3p	0.93	0.042	**hsa-miR-194-5p**	0.25	0.031
**hsa-miR-15a-5p**	0.91	0.003	hsa-miR-30b-5p	0.25	0.004
hsa-miR-10b-5p	0.88	0.042	**hsa-miR-140-3p**	0.24	0.031
**hsa-miR-92a-3p**	0.83	0.003	hsa-miR-29b-3p	0.24	0.028
**hsa-miR-101-3p**	0.78	0.011	**hsa-miR-93-5p**	0.23	0.032
hsa-miR-20b-5p	0.75	0.044	**hsa-miR-92a-3p**	0.23	0.028
hsa-miR-210	0.74	0.012	hsa-miR-484	0.21	0.014
hsa-miR-497-5p	0.73	0.042	hsa-let-7g-5p	0.21	0.014
**hsa-miR-19b-3p**	0.72	0.018	hsa-miR-148b-3p	0.2	0.017
hsa-miR-30a-5p	0.66	0.03	hsa-miR-30e-5p	0.2	0.01
**hsa-miR-19a-3p**	0.61	0.019			
**hsa-miR-15b-3p**	0.59	0.036			
**hsa-miR-425-5p**	0.42	0.031			
**hsa-miR-93-5p**	0.37	0.042			
**hsa-miR-20a-5p**	0.35	0.046			

miRNAs downregulated upon hydromorphone and/or oxycodone treatment are presented. Underlined miRNAs are commonly downregulated by both hydromorphone and oxycodone treatment. *q*-values are FDR adjusted *p* values of paired *t*-test between pre and 24 h.

## Abbreviations

CNS	central nervous system
ER	extended release
miRNA	microRNA
MOR	μ-opioid receptor
PCR	polymerase chain reaction
qRT-PCR	quantitative real-time PCR
UTR	untranslated region
